# Inhibitory Effect of a Microecological Preparation on Azoxymethane/Dextran Sodium Sulfate-Induced Inflammatory Colorectal Cancer in Mice

**DOI:** 10.3389/fonc.2020.562189

**Published:** 2020-10-16

**Authors:** Weinan Yu, Jie Zhang, Zhewen Chen, Shuai Wang, Chuanxian Ruan, Wenli Zhou, Mingyong Miao, Hanping Shi

**Affiliations:** ^1^Departments of Endocrinology, The Affiliated Huai’an Hospital of Xuzhou Medical University, Huai’an, China; ^2^Department of Nutrition, Zhejiang Provincial People’s Hospital, Hangzhou, China; ^3^Departments of Gastroenterology, The Affiliated Huai’an Hospital of Xuzhou Medical University, Huai’an, China; ^4^Microbial Reserch Institute, Japan Kyowa Industrial Co., Ltd., Tokyo, Japan; ^5^Department of Medical Oncology, Changzheng Hospital, Navy Medical University, Shanghai, China; ^6^Department of Biochemistry and Molecular Biology, The Naval Medical University, Shanghai, China; ^7^Department of Gastrointestinal Surgery, Department of Clinical Nutrition, Beijing Shijitan Hospital, Capital Medical University, Beijing, China

**Keywords:** colorectal cancer, microecological preparation, azoxymethane/dextran sodium sulfate, intestinal microbiota, metabolites

## Abstract

This study aims to investigate the antitumor effect and the possible mechanism of a microecological preparation (JK5G) in mice. The mice treated with AOM/DSS were then randomly divided into the two model groups and the JK5G group, and the blank control group was included. Fecal samples were used for liquid chromatography–mass spectrometry and 16S rRNA gene sequencing analyses to reveal metabolic perturbations and gut flora disorders to demonstrate the effects of JK5G. Compared with the mice in the control group, the weight and food intake of mice after JK5G treatment were both upregulated. Moreover, JK5G could inhibit the growth of colon tumors and prolong the survival rate of mice, as well as inhibit the levels of cytokines in serum. The proportions of lymphocytes, T cells, CD3^+^CD4^+^ T cells, and CD3^+^CD8^+^ T cells in the spleen of the JK5G mice were all significantly increased compared to those in the control group (*p* < 0.05). Similarly, compared with the model group, the proportions of lymphocytes, B cells, T cells, natural killer T cells, CD3^+^CD4^+^ T cells, and CD3^+^CD8^+^ T cells in the intestinal tumors of the JK5G mice were significantly increased (*p* < 0.05). Furthermore, 16S rRNA high-throughput sequencing data revealed that *Alloprevotella* in the JK5G group was significantly upregulated, and *Ruminiclostridium*, *Prevotellaceae_UCG_001*, and *Acetitomaculum* were significantly downregulated. Fecal and serum metabolite analysis detected 939 metabolites, such as sildenafil and pyridoxamine, as well as 20 metabolites, including N-Palmitoyl tyrosine and dihydroergotamine, which were differentially expressed between the JK5G and model groups. Integrated analysis of 16s rRNA and metabolomics data showed that there were 19 functional relationship pairs, including 8 altered microbiota, such as *Ruminiclostridium* and *Prevotellaceae_UCG_001*, and 16 disturbed metabolites between the JK5G and model groups. This study revealed that JK5G treatment was involved in the growth of colorectal cancer, which may be associated with the role of JK5G in improving the nutritional status of mice and regulating the tumor microenvironment by regulating the changes of intestinal microbiota and metabolite bands on different pathways.

## Introduction

Intestinal flora is one of the most complex microbial systems in the human body. It is in a dynamic balance of symbiosis, coexistence, and co-prosperity between human hosts, and has a very important physiological function for human health (1, 2). More and more studies have found that intestinal flora plays an important role in the occurrence of tumors, while digestive system tumors, as one of the most common malignant tumors in the world, also pose a serious threat to human health and constitute a major source of economic burden in the field of world public health (3, 4). Colorectal cancer (CRC), the most common malignancies of digestive system, is the fourth deadliest cancer in the world, accounting for about 881,000 deaths worldwide in 2018. Most CRCs are characterized by an orderly carcinogenic process in which mutations accumulate over an average of 10 to 15 years (5, 6).

In recent years, the clinical application of microecological preparations in CRC has been increasing gradually (7). In the perioperative period of CRC, intestinal microecological environment disorders (intestinal flora imbalance, intestinal barrier function damage, and bacterial flora migration) and cellular immune function decline often occur, which is not conducive to postoperative intestinal function recovery, while cellular immune function decline provides an opportunity for tumor recurrence (3, 8, 9). However, some microecological nutrition agents can promote the recovery of intestinal mucosal barrier function, reduce the incidence of postoperative complications, and shorten the length of hospital stay in patients with CRC during the perioperative period. Usually, the microecological balance can be adjusted and maintained through enzyme action, antibacterial action, adhesive colonization, and biological barrier, while improving the health of the host. Previous studies have reported that the main functions of microecologics can be summarized as protection, immunity, bacteriostasis, balance, nutrition, antitumor, liver protection, and downregulating blood glucose.

With the development of high-throughput sequencing technology, many studies have shown that microbial metabolites have important effects on host physiology. Notably, 16S rRNA sequencing technique is a fast and well-tested method widely used to analyze the differential abundance of microbial communities and their correlation with environmental factors (10, 11). Moreover, based on liquid chromatography–mass spectrometry (LC–MS), metabolomics are emerging as valuable tools for the targeted profiling of numerous small molecular metabolites. Intestinal dysfunction has the effects on the body’s absorption and metabolism, resulting in metabolic disorders. The combination of microbiological and metabolomic techniques suggests that the imbalance of intestinal flora is directly related to the imbalance of various metabolites in many diseases (12–14).

The azoxymethane/dextran sodium sulfate (AOM/DSS) mouse model stands as a relevant preclinical inflammation-associated CRC model with histological and phenotypic features. AOM/DSS simulates the physiological and pathological process of cancer induced by chronic intestinal inflammation, and therefore, it has been widely used to study the formation mechanism of inflammation-related cancer (15, 16). The purpose of this study was to study the antitumor effect of a microecological preparation, JK5G, by constructing the tumor model.

## Materials and Methods

### Animals and Experimental Design

Thirty specified pathogen-free C57BL/6J mice (male, aged 4–5 weeks) were obtained from Changzhou Cavens Laboratory Animal Co. Ltd. All animals were housed with free access to water and food under standard laboratory conditions. A total of 20 mice were randomly selected to generate the AOM/DSS model of CRC. Furthermore, the mice treated with AOM/DSS were then randomly divided into the two groups, including model and JK5G groups. Briefly, the mice were injected intraperitoneally with AOM (10 mg/kg). A week later, DSS drinking water was prepared and the molecular weight was 36,000–50,000 DSS dissolved in sterilized water, configured as 2.5% DSS solution, and then the mice were provided regular sterile water for 14 days. All the water intake was not limited. Additionally, another 10 mice were set as the control group and were injected with an equal volume of normal saline intraperitoneally. The JK5G group was administered 10 mg once a day on the first injection of AOM (Japan Kyowa Industrial Co., Ltd.) through oral gavage. Microecological preparation JK5G is a high-concentration complex that is rich in bacteria and their metabolites, including lactococcus and 21 kinds of compound lactobacillus bacteria and peptidoglycans and metabolites from lactococcus cytoderm.

At 0 and 8 weeks, fecal samples were collected and then stored at −80°C before the analysis of metabolic profiling and microbial community. The animal protocol was approved by the Animal Ethics Committee of Xuzhou Medical University. At the end of the experimental procedure, 10 mice from each group were randomly selected for the detection of hind leg muscle circumference. Additionally, the level of albumin in serum was quantified using ELISA kits (R&D Systems Europe, United Kingdom) in accordance with the instructions provided by the manufacturer. Moreover, mice were anesthetized by intraperitoneal injection of 3% sodium pentobarbital (40 mg/kg). Then, the blood, spleen, and intestinal tissue samples were collected for data analysis. All samples were stored at −80°C until additional analyses.

### Extraction of Single-Cell Suspension From Intestinal Tumor or Spleen

To extract single cells from intestinal tumors, 1–2 cm of cecum tumor tissue was placed in 10 ml of 1640 medium containing 10% fetal bovine serum. After that, the tissue samples were added to solution A [50 ml of PBS, 3 ml of 0.5 mM ethylenediaminetetraacetic acid, 500 μl of 1 M 4-(2-hydroxyethyl)-1-piperazineethanesulfonic acid, and 25 μl of 2 M dithiothreitol] and shaken vigorously at 37°C for 10 min to remove intestinal epithelial cells. Subsequently, the tissue samples were transferred to a new 50-ml centrifuge tube filled with 10 ml of liquid B [50 ml of PBS, 3 ml of 0.5 mM ethylenediaminetetraacetic acid, and 500 μl of 1 M 4-(2-hydroxyethyl)-1-piperazineethanesulfonic acid] and shaken vigorously for 10 min. Following washing with 10% fetal bovine serum, 200 U/ml and 1 mg/ml type VIII collagenase was added for 1–1.5 h. The cell precipitate was resuspended with 4 ml 40% percoll solution and 2.5 ml of 80% percoll solution.

To extract single cells from spleen tissues, the spleen was ground with the blunt end of the syringe, and the cell suspension was absorbed by the dropper and filtered through a 100-μm filter into a 50-ml centrifuge tube. After centrifugation, the supernatant was removed, and 2 ml of erythrocyte lysis buffer was added at room temperature for 2 min. Then, 2 ml of PBS solution was added and centrifuged at 400 *g* for 5 min to remove the supernatant, which was repeated twice to obtain the single-cell spleen suspension.

### ELISA

The levels of inflammatory factors, including tumor necrosis factor (TNF)-α, interleukin (IL)-2, IL-4, IL-6, IL-10, and interferon-γ in the serum, were measured using commercial mouse ELISA kits (Quanzhou Konodi Biotechnology Co., Ltd.). The absorbance of the final products was detected on a microplate reader at the wavelength of 450 nm.

### Flow Cytometry

The cells were harvested from the mouse spleen and tumor tissues, with 10% PBS and 0.5 mM EDTA. Digested tissues were centrifuged at 2000 rpm for 7 min and resuspended using 40% percoll (GE, 17-0891-09). The cells were centrifuged at 2500 rpm for 20 min again and then incubated with antibodies (the exact antibody information was shown in the Supplementary Table S1). The BD LSRFortessa X-20 (Biosciences, San Jose, CA, United States) was used for flow cytometry analysis. CD4^+^CD25^+^Foxp3^+^ characterization was performed according to the eBiosciences kit (San Diego, CA, United States).

### Histological Examination

The tissues samples were fixed in neutral formalin [10% (v/v)], embedded in paraffin, and sectioned into 5-μm slices. Then, the sections were stained with hematoxylin and eosin, and the infiltration of immune cells in tissue sections was examined under light microscopy.

### DNA Extraction and Sequencing

The microbial DNA was extracted from 50 mg of thawed fecal sample by EZNA stool DNA kit. By using the diluted genomic DNA as the template and based on the selection of the sequencing region, the specific primer with Barcode and Takara Ex Taq high-fidelity enzyme of Takara Company were used for PCR to ensure the amplification efficiency and accuracy. The V3 and V4 hypervariable region of the 16S rRNA gene was amplified with polymerase chain reaction by employing forward (5′-TACGGRAGGCAGCAG-3′) and reverse primers (5′-AGGGTATCTAATCCT-3′). Purified libraries were constructed following the manufacturer’s instructions, and Illumina MiSeq platform (Illumina, San Diego, CA, United States) was used for sequencing. There were six samples for each group.

USEARCH software was used to demultiplex the raw read (17). Operational taxonomic unit (OTU) picking was analyzed with Quantitative Insights Into Microbial Ecology bioinformatics pipeline (18). The gene sequences of 16S rRNA were clustered at 97% similarity using UCLUST (17). The indexes of alpha diversity were calculated with MOTHUR (3). The rarefaction curve and bar graphs were drawn with the R vegan package (19). Beta-diversity was estimated and visualized using principal coordinate analysis.

To predict metagenomic function, 97% of OTUs were selected with closed-reference OTU selection protocol (Quantitative Insights Into Microbial Ecology), as well as the Greengenes database (20). Reconstruction of the metagenome was conducted by Phylogenetic Investigation of Communities by Reconstruction of Unobserved States software (21). Predicted functional genes were cataloged into the KEGG orthology and then compared between different groups (model vs. control, JK5G vs. model) using Kruskal–Wallis tests. The pathway with *p* < 0.05 was considered significant.

### LC-MS Metabolomics Processing

Fecal samples for metabolomics were analyzed using the LC-MS platform. The UPLC system was equipped with an AB Sciex Triple TOF 5600 mass spectrometer. Briefly, the samples were thawed at room temperature, and then each sample was transferred to a 1.5-ml centrifuge tube. After adding 10 μl of internal standard, each sample was vortexed for 30 s, and then centrifuged at 12,000 rpm at 4°C for 15 min. Sequentially, the supernatant (200 μl) was transferred to a new bottle. Mass spectrometer (MS) detection was conducted with the quadrupole time-of-flight mass spectrometry system in electrospray ionization in positive ion mode, and the specific conditions were as follows: desolvation temperature, 500°C; de-cluster voltage, 80 eV; ion spray voltage, 5500 V; collision energy, 10 eV; and desolvent gas flow rate, 50 ml/min.

### Metabolomics Data Analysis

In order to complete the data processing of metabolomics, principal component analysis, partial least squares discriminant analysis, and orthogonal partial least squares discriminant analysis were conducted on the raw data of LC-MS. At the beginning of the analysis, quality control samples were injected and tested every five samples to assess LC-MS stability throughout the collection process. The characteristics of less than 50 or 80% of the biological samples detected in the quality control samples were removed. The ions were identified by combining the retention time and *m*/*z* data.

The significant differences between groups (model vs. control, JK5G vs. model) in metabolites were analyzed by Student’s *t* tests, with a *p*-value < 0.05 and Vip_oplsda > 1 considered to indicate statistical significance. Metabolic pathway analysis was conducted for differentially expressed metabolites that exhibited significant differences using the Kyoto Encyclopedia of Genes and Genome database^1^. The *p*-value was adjusted for multiple tests (Benjamini–Hochberg), and a *p*-value < 0.05 was considered to be statistically significant.

### Integrated Analysis of 16s rRNA and Metabolomics Data

In order to detect the relationships between the gut microbiota and their potential association with metabolites, the common or similar pathways were selected. Furthermore, co-expression relationship pairs of 16S rRNA and metabolomics data in the model versus control groups and the JK5G versus model groups, respectively, were screened. The relationship pairs with correlation coefficients |*r*| > 0.8 and *p* < 0.001 were used for exploring the correlation of fecal microbiota and metabolites.

### Statistical Analysis

SPSS 19.0 and GraphPad Prism 7.0 were used for statistical analysis. Survival analysis was conducted by the Kaplan–Meier method. The differences between two groups were evaluated with the two-tailed Student’s *t*-test, and the differences among three groups were analyzed using one-way analysis of variance.

## Results

### General Observation

**FIGURE 1:**
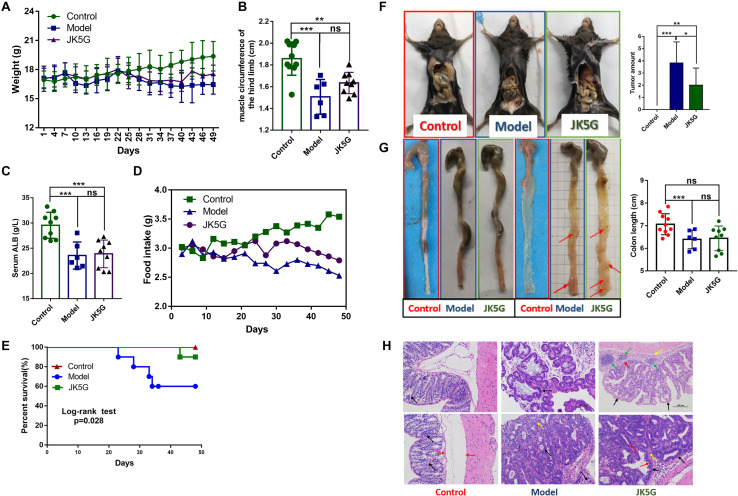
Mouse weights, general nutritional status, food intake, and survival rate and growth of colorectal cancer over time. When compared with the model group, the weight **(A)**, food intake **(D)**, and survival rate **(E)** of the microecological preparation group were significant (*p* < 0.05). The muscle circumference **(B)** and serum albumin **(C)** of the hindlimb of the microecological preparation group were higher than those of the model group, but the difference was not significant (*p* > 0.05). Compared with the model groups, the tumor size **(F)** and number **(G)** were lower in the microecological preparation group (*p* < 0.05). Hematoxylin and eosin staining of colon tissues showed that the tissue in the model group exhibited an obvious boundary between the tumor and surrounding tissues, with inflammatory cells infiltrating into the submucosa **(H)**. *p < 0.05; **p < 0.01; ***p < 0.001.

As shown in Figure 1A, after 25 days of intervention, the weight of mice in the control group increased slowly, but the weight of mice in the model group and the JK5G group decreased gradually. Moreover, the weight of mice in the JK5G group was always slightly higher than that in the model group. At the end of the experiment, the weight of the control, model, and JK5G groups was 19.38 ± 1.52, 16.44 ± 0.99, and 17.56 ± 0.91 g, respectively, and the differences were statistically significant. In addition, when compared with the control group, the muscle circumference and serum albumin levels of the hind limbs of mice in the model group and the JK5G group were significantly decreased (Figures 1B,C). The muscle circumference of the hindlimb of the JK5G group was higher than that of the model group; however, the difference was not significant.

Additionally, at 3 weeks of the experiment, the food intake of mice in the model group and the JK5G group decreased significantly; however, the food intake of mice in the JK5G group was always upregulated in comparison to that in the model group (Figure 1D).

Furthermore, during the intervention period, no mice died in the control group (0/10), four mice died in the model group (4/10), and one mouse died in the JK5G group (1/10). The mortality rates of mice in the three groups were 0, 40, and 10%, respectively. Survival analysis showed that there were observably differences in survival rates in three groups (*p* = 0.028; Figure 1E).

### JK5G Could Inhibit the Growth of Colon Tumor

The colon tissue was collected from mice at the end of the study, and tumor growth was observed. The results showed that there was an observable difference in colon tumor number among the three groups (*p* < 0.001; Figure 1F). The mean number of colon tumors in the JK5G group was observably lower than that in the model group (*p* < 0.05), suggesting that JK5G could inhibit the growth of colon tumors. Furthermore, the colon of mice in the model group was observably shorter than that in the control group (*p* < 0.001; Figure 1G). However, there was no observable difference in colonic length between the JK5G group and the model group.

More importantly, at the middle of the experiment, hematoxylin and eosin staining of the colon tissues showed that there was no normal intestinal gland structure in the intestinal mucosa of the model group and JK5G group, but irregular tubular and focal hyperplasia of glands formed by the arrangement of tall columnar tumor cells and protruding into the lumen (black arrow). However, the structural damage of intestinal glands in the model group was more serious than that in the JK5G group. Moreover, at the end of the experiment, tumor tissues of the colon in the model group and the JK5G group grew in compression to the surrounding tissues, which were clearly separated from the surrounding tissues. Meanwhile, there was a small amount of mitosis (red arrow) and a small amount of inflammatory cell infiltration in interstitial connective tissue (yellow arrow) (Figure 1H). All these data further confirmed that JK5G could inhibit the growth of colon tumors induced by AOM/DSS treatment.

### JK5G Reduces Serum IL-6, IL-10, and TNF-α in AOM/DSS-Treated Mice

**FIGURE 2:**
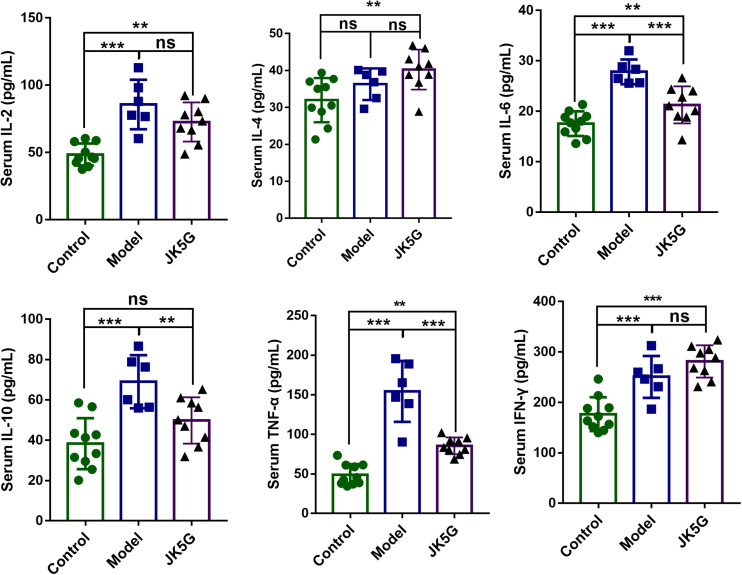
Microecological preparation reduces interleukin (IL)-6, IL-10, and tumor necrosis factor-alpha (TNF-α) levels in AOM/DSS-treated mice. Compared with the control group, the IL-6, IL-10, and TNF-α levels were significantly reduced compared to those in the model group. *p < 0.05; **p < 0.01; ***p < 0.001.

To further detect the effects of JK5G on CRC carcinogenesis, the serum cytokines levels were measured by ELISA. As illustrated in Figure 2, when compared with the control group, the IL-2, IL-4, IL-6, IL-10, TNF-α, and interferon-γ in the model group were all upregulated. Furthermore, TNF-α, IL-2, and IL-6 levels in the JK5G group were observably reduced in comparison to those in the model group (*p* < 0.05).

### JK5G Regulates Immune Cells in AOM/DSS Mice

**FIGURE 3:**
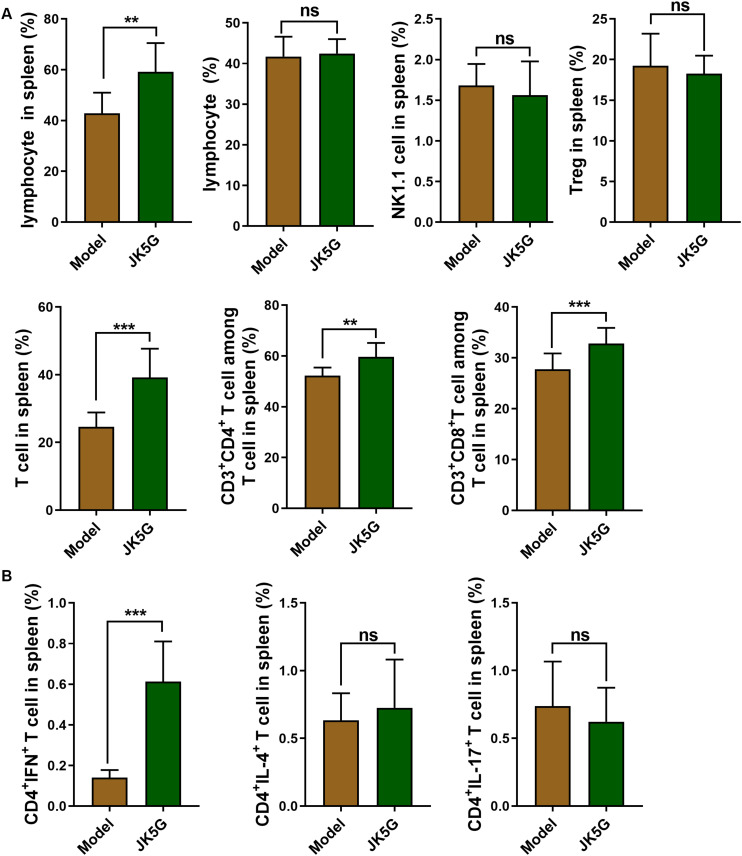
Flow cytometry of the immune cells in spleen. Compared with the model group, the proportions of lymphocytes, T cells, CD3^+^CD4^+^ T cells, and CD3^+^CD8^+^ T cells **(A)**, as well as CD4^+^interferon^+^ T cells **(B)** in the microecological preparation group were significantly increased (**p < 0.01; ***p < 0.001).

Subsequently, the immune cells in the spleen including lymphocytes, B cells, natural killer cells, and T cells, as well as helper T cells (CD3^+^CD4^+^ T cells), cytotoxic T cells (CD3^+^CD8^+^ T cells), and regulatory T cells (Treg T cells), were further investigated. The results revealed that in comparison to the model group, the proportions of lymphocytes, CD3^+^CD4^+^ T cells, and CD3^+^CD8^+^ T cells in the JK5G group were observably upregulated (Figure 3A and Supplementary Figure S2; all, *p* < 0.05). In addition, the proportions of CD4^+^interferon^+^ T cells in spleen of mice in the JK5G group were observably higher than those in the model group (*p* < 0.001; Figure 3B and Supplementary Figure S3).

**FIGURE 4:**
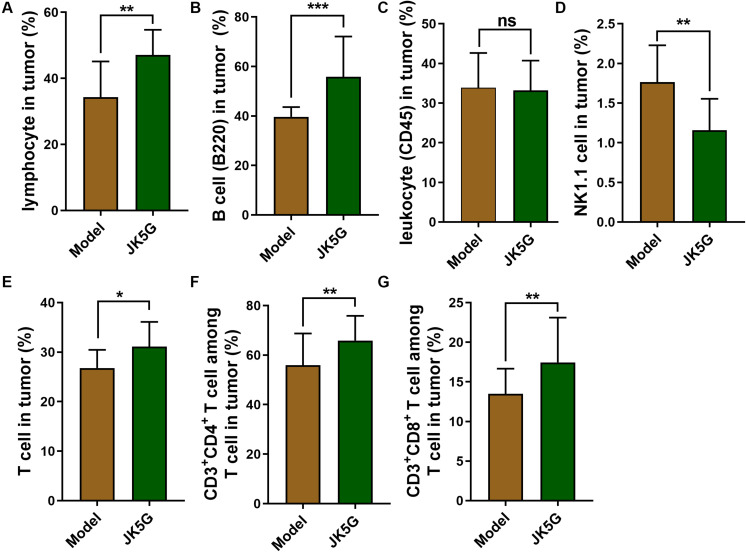
The comparison of proportions of lymphocytes **(A)**, B cells **(B)**, leukocyte **(C)**, natural killer T cells **(D)**, T cells **(E)**, CD3^+^ CD4^+^T cells **(F)** and CD3^+^ CD8^+^T cells **(G)** between Model group and JK5G group in tumor. *p < 0.05; **p < 0.01; ***p < 0.001.

Likewise, the proportions of lymphocytes, B cells, total T cells, CD3^+^CD4^+^ T cells, natural killer T cells, and CD3^+^CD8^+^ T cells in the spleen and tumor tissues of the JK5G group were observably upregulated compared to those in the model group (all *p* < 0.05; Figure 4).

### JK5G-Induced Changes in the Intestinal Bacterial Microbiome

The results of alpha diversity analysis showed that the species accumulation curve in this experiment tended to be flat, indicating the adequacy of sampling (Supplementary Figures S1A–C); furthermore, according to Supplementary Figures S1D, S2F, the samples of the same group were basically gathered together, indicating that the differences within the group were small and the experiment was reliable.

**FIGURE 5:**
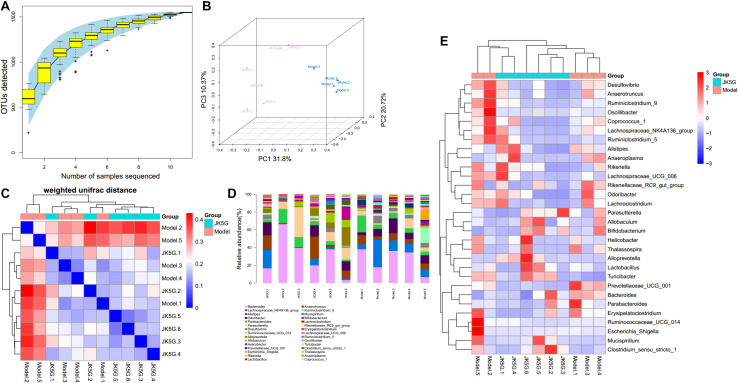
Different flora analysis on the model versus control groups and the microecological preparation versus model groups. Specaccum species accumulation curve **(A)**, principal component analysis **(B)**, β diversity index **(C)**, the bluer the color, the closer the distance between the samples, the higher the similarity, and the farther the red color; histogram of colony composition **(D)**, the heat map of community composition **(E)**.

Different flora analysis was conducted on the model versus control groups and the JK5G versus model groups at the phylum and genus levels, respectively (Figure 5). The significant differences were detected using a Kruskal–Wallis test, and the flora with *p*-value < 0.05 is generally considered to be a significantly different flora. As shown in Supplementary Table S2A, the phylum Verrucomicrobia and Actinobacteria were overrepresented in the model group compared to the control group. At the genus level, the 16s rRNA expression of *Prevotellaceae_UCG_001*, Bifidobacterium, and Akkermansia was observably increased in the model group (*p* < 0.05) in comparison to those in the control group, while Lachnospiraceae_UCG_008, Ruminococcaceae_UCG_005, and Lachnospiraceae_UCG_001 levels were observably downregulated in the model group. Similarly, Supplementary Table S2B showed the top 10 differential microbiome between the JK5G and model groups. *Alloprevotella* was found to be overrepresented in JK5G group as compared to the model group. Conversely, the 16s rRNA levels of *Ruminiclostridium*, *Prevotellaceae_UCG_001*, and *Acetitomaculum* in JK5G group were observably downregulated compared to those in model group (all *p* < 0.05).

Using Phylogenetic Investigation of Communities by Reconstruction of Unobserved States software, the different pathways between the model versus control groups, as well as the JK5G versus model groups, were screened. Briefly, a total of 20 pathways (Supplementary Table S3) and 7 pathways, such as ether lipid metabolism and linoleic acid metabolism, were found to be different, respectively. More importantly, as illustrated in Figure 5, the different pathways could be significantly distinguished between the two groups.

### JK5G-Induced Gut Fecal Metabolic Profiling Analysis

**FIGURE 6:**
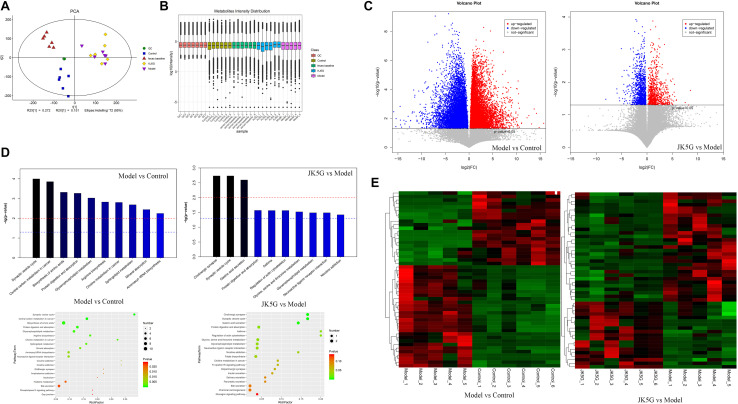
Different metabolites in feces on the model versus control groups, and the microecological preparation (JK5G) versus model groups. Principal component analysis **(A),** boxplot of metabolite strength **(B)**, volcano plot of different metabolites in the model versus control groups, and the JK5G versus model groups **(C)**, enrichment pathways of differential metabolites **(D)**, heat map clustering of different metabolites in the model versus control groups, and the JK5G versus model groups **(E)**.

Principal component analysis showed that the stool metabolite quality control samples were closely clustered together, indicating that the experiment was stable and reproducible (Figures 6A,B). A total of 172 differentially expressed metabolites (including 106 downregulated and 66 upregulated) were obtained between the model and control groups, such as carboxylic acid, 3-galactosyllactose, indole, lenticin, bassic acid, and so on. Moreover, 939 metabolites (including 423 downregulated and 517 upregulated) were found to be differentially expressed between the JK5G and model groups (Figures 6C,E) including pyridoxamine, stevioside, isoleucyl-tyrosine, dinorcapsaicin, 2-hydroxycinnamic acid, L-valine, serotonin, and so on. The top 20 differentially expressed metabolites are summarized in Supplementary Table S4, respectively.

To further explore the mechanism of action of the differentially expressed metabolites, a KEGG database pathway analysis was performed. According to Figure 6D, 30 pathways were enriched between the model and control groups, such as synaptic vesicle cycle, central carbon metabolism in cancer, biosynthesis of amino acids, choline metabolism in cancer, mineral absorption, and so on, and 11 pathways were enriched by the differentially expressed metabolites between the JK5G and model groups, including cholinergic synapse, synaptic vesicle cycle, gastric acid secretion, regulation of actin cytoskeleton, and so on.

### JK5G-Induced Serum Metabolic Profiling Analysis

**FIGURE 7:**
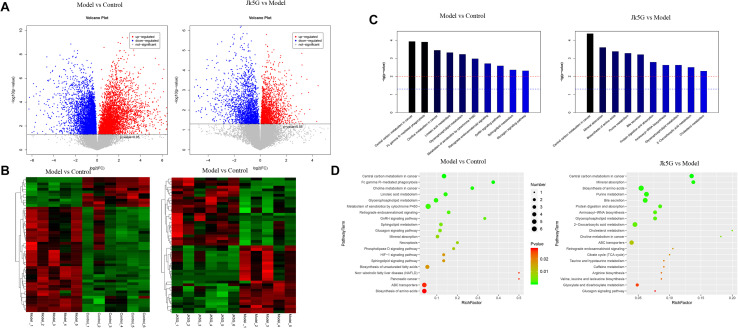
Different metabolites in serum on the model versus control groups, and the microecological preparation (JK5G) versus model groups. Volcano plot of different metabolites in the model versus control groups, and the JK5G versus model groups **(A)**, heat map clustering of different metabolites in the model versus control groups, and the JK5G versus model groups **(B)**, enrichment pathways of differential metabolites **(C,D)**.

Twenty differentially expressed metabolites were obtained between the model and control groups (Supplementary Table S5), such as hydroxydecanoic acid, kynurenine, 11Z-hexadecenoic acid, dehydrovomifoliol, noravicholic acid, pyranomammea c, and so on. Moreover, 20 metabolites were found to be differentially expressed between the JK5G and model groups, including N-palmitoyl tyrosine, dihydroergotamine, liquoric acid, sulfacetamide, kolanone, eszopiclone, glucoside, flutamide, L-urobilin, and so on. Cluster analysis showed that these differentially expressed metabolites could be distinguished significantly in different groups (Figures 7A,B). Furthermore, according to Figures 7C,D, 26 pathways, such as central carbon metabolism, linoic acid metabolism, glucagon signaling pathway, and choline metabolism in cancer, were enriched between the model and control groups, and 28 pathways were enriched by the differentially expressed metabolites between the JK5G and model groups, including central carbon metabolism in cancer, mineral absorption, biosynthesis of amino acids, bile secretion, and so on.

Furthermore, compared to the results of fecal metabolic pathways, there were 10 common pathways, such as central carbon metabolism in cancer, choline metabolism in cancer, glycerophospholipid metabolism, and sphingolipid metabolism, in the model versus control groups and two common pathways (protein digestion and absorption and glycerophospholipid metabolism) in the JKG5 versus model groups.

### Correlation Between the Gut Microbiome and Metabolome

To further explore the relationship between the metabolites and intestinal flora, the common or similar pathways were screened according to the integrated analysis. In the model versus control groups, the results showed sphingolipid metabolism, sphingolipid signaling pathway, and glycosphingolipid biosynthesis; the lacto and neolacto series pathway affected the disease at the metabolomic level, but it is also closely related to the disease in the intestinal flora. However, there were no pathways with the same or similar functions between the JK5G and model groups through the integrated analysis of 16s rRNA and the metabolome.

**FIGURE 8:**
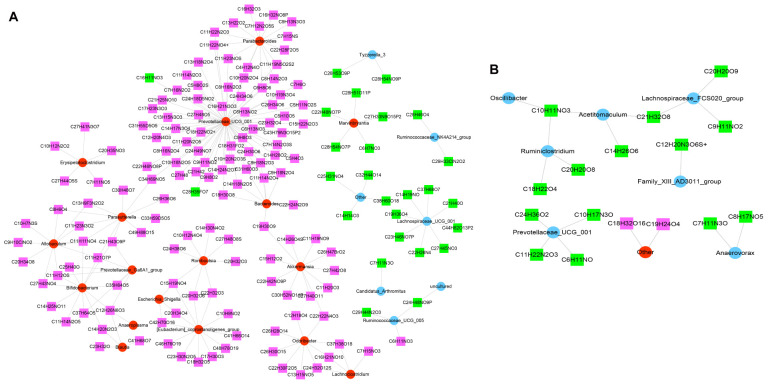
Co-expression of differential microbiota and metabolites. Model versus control **(A)** and microecological preparation versus model **(B)**. The red circle is upregulated differential bacteria, the blue circle is downregulated differential bacteria, the green square represents downregulated differential metabolites, and the pink square represents upregulated differential metabolites.

According to the Spearman correlation coefficient of |*r*| > 0.8 (*p* < 0.001), 209 relationship pairs, including 24 different microbiota and 159 different metabolites, were screened in the model versus control (Figure 8A). In addition, Figure 8B revealed that there were 19 functional relationships pairs, including eight altered microbiota, such as *Ruminiclostridium* and *Prevotellaceae_UCG_001*, and 16 disturbed metabolites, such as C10H11NO3 and C21H32O8, following JK5G application.

## Discussion

Despite the advancement in the therapeutic strategies of CRC, the long-term prognosis of patients with the disease remains unsatisfactory; therefore, it is still urgent to identify useful drugs without side effects for the treatment and prognosis of CRC. This study based on the AOM/DSS-derived CRC mice model indicated that JK5G suppresses the growth of CRC, resulting in the better survival rate of CRC patients; moreover, JK5G treatment has significant effects on intestinal microbiota and metabolites by regulating the different pathways involved in CRC.

The combination of microecologics and intestinal mucosa can prevent the reproduction and invasion of bacteria to strengthen the barrier function of the intestinal mucosa and the antibacterial effect. Medina et al. (22) have found that Bifidobacterium and Lactobacillus have the function of regulating immunity, and some living bacteria and their metabolites can induce the production of interferon, enhance the body’s immunity, and play antitumor and anti-allergy roles. A study has found that adding beneficial bacteria to traditional enteral nutrition can inhibit the growth of pathogenic bacteria, improve the distribution of intestinal flora, and improve the body’s immunity and anti-infection ability (23). In the present study, the results showed that JK5G treatment could significantly inhibit the growth of colon tumors, as well as the levels of cytokines in serum, such as IL-2, IL-6, and TNF-α. Moreover, the proportions of lymphocytes, T cells, CD3^+^CD8^+^ T cells, and CD3^+^CD4^+^ T cells in the JK5G group were observably upregulated compared to those in the control group. Usually, plasma TNF-α, IL-2, and IL-6 have been regarded as important cytokines related to the progression of CRC (24, 25). Moreover, CRC is associated with increased concentrations of IL-2, IL-6, and TNF-α. Furthermore, the increase of Tregs, lymphocytes, T cells, and CD3^+^CD4^+^ T cells have been widely observed in CRC patients (26, 27). The data suggested that JK5G treatment could suppress the progression of CRC. In this study, CD4^+^ and CD8^+^T cells were increased while Treg and NK were not changed. We guess NK and Treg were insignificantly decreased in JK5G, which may raise the possibility of combined hyperglycemia of CD4^+^ and CD8^+^T cells.

Microecologics have been used in feed, agriculture, medicine, health care, and food. In the future, a pollution-free preparation following the natural circulation law of the ecological environment, microecologics will be a development trend in the additive industry. The gut flora is composed of different bacteria that produce a wide variety of metabolites. It is specifically responsible for the selection of microorganisms and plays important roles in metabolic functions (28). Factors such as nutritional intervention, host condition, as well as toxicological injury can induce microbial regulation and affect host health. Hence, comprehensive analysis of intestinal flora and metabolites is helpful to understand the changes of host physiological state under the above interference types (29). In the present study, we found that *Alloprevotella* was found to be overrepresented, but *Ruminiclostridium*, *Prevotellaceae_UCG_001*, and *Acetitomaculum* in the JK5G group were observably downregulated in comparison to those in the model group. Furthermore, the integrated analysis of 16s rRNA and metabolomics data showed that there were 19 functional relationship pairs, including eight altered microbiota (e.g., *Ruminiclostridium* and *Prevotellaceae_UCG_001*) and 16 disturbed metabolites (e.g., C10H11NO3 and C21H32O8) between the JK5G and model groups. Actually, *Alloprevotella* has been regarded as the cancer-related bacteria in colon cancer or visceral leishmaniasis (30, 31). Moreover, a previous study showed that the abundance of the *Ruminiclostridium* genus was related to the enhancement of animal immune responses to spore adsorbent antigen and probiotics (32). Similarly, *Prevotellaceae_UCG_001* was overrepresented in AOM/DSS mice in comparison to controls (33). Hence, we speculate that microecologics play a role in regulating tumor microenvironment (immunity and inflammation) through intestinal flora and its metabolites. More importantly, pathway analysis in this study revealed several differential pathways such as ether lipid metabolism and linoleic acid metabolism pathways. In fact, ether lipid metabolism has been reported to be found in oral squamous cell carcinoma (34); moreover, linoleic acid metabolism was found to play an important role in hepatocarcinogenesis (35). However, their role activated by the intestinal bacteria in CRC remains unclear and whether the microecologics affect intestinal flora and metabolites by regulating these pathways needs to be verified in a future study.

In conclusion, JK5G inhibiting the development of CRC may be associated with improving the nutritional status of mice and regulating the tumor microenvironment (immunity and inflammation) by modulating the proportions of intestinal microbiota and metabolites.

## Data Availability Statement

The datasets presented in this study can be found in online repositories. The names of the repository/repositories and accession number(s) can be found in the article/ Supplementary Material.

## Ethics Statement

The animal study was reviewed and approved by The Animal Ethics Committee of Xuzhou Medical University. Written informed consent was obtained from the owners for the participation of their animals in this study.

## Author Contributions

HS, MM, WY, WZ, and CR conceived and designed the experiments. JZ, ZC, and SW performed the experiments and analyzed the data. JZ and WY drafted and revised the manuscript. All authors read and approved the final manuscript.

## Conflict of Interest

CR was employed by Japan Kyowa Industrial Co., Ltd. The remaining authors declare that the research was conducted in the absence of any commercial or financial relationships that could be construed as a potential conflict of interest.

## Footnotes

1 https://www.kegg.jp/

## Supplementary Material

Supplementary Figure 1 Diversity analysis. Alpha diversity analysis **(A–C),** the horizontal axis is sample size, and the vertical axis is the number of OTUs. If the curve shows a sharp rise, it indicates that the sampling quantity is insufficient as the sampling quantity increases; if the curve tends to flatten, it means that the species in this environment will not increase significantly with the increase of sample size, indicating that the sampling is adequate. Beta-diversity analysis **(D–F),** the horizontal and vertical axes of the figure represent two eigenvalues that can reflect the variance to the greatest extent. Each point in the figure is a sample, and the same color is the same group.
