# Supplementary figures and images for: Inhibitory Effect of a Microecological Preparation on Azoxymethane/Dextran Sodium Sulfate-Induced Inflammatory Colorectal Cancer in Mice
Source: Front Oncol. 2020 Oct 16;10:562189. doi: 10.3389/fonc.2020.562189 (PMC7596756; doi:10.3389/fonc.2020.562189)

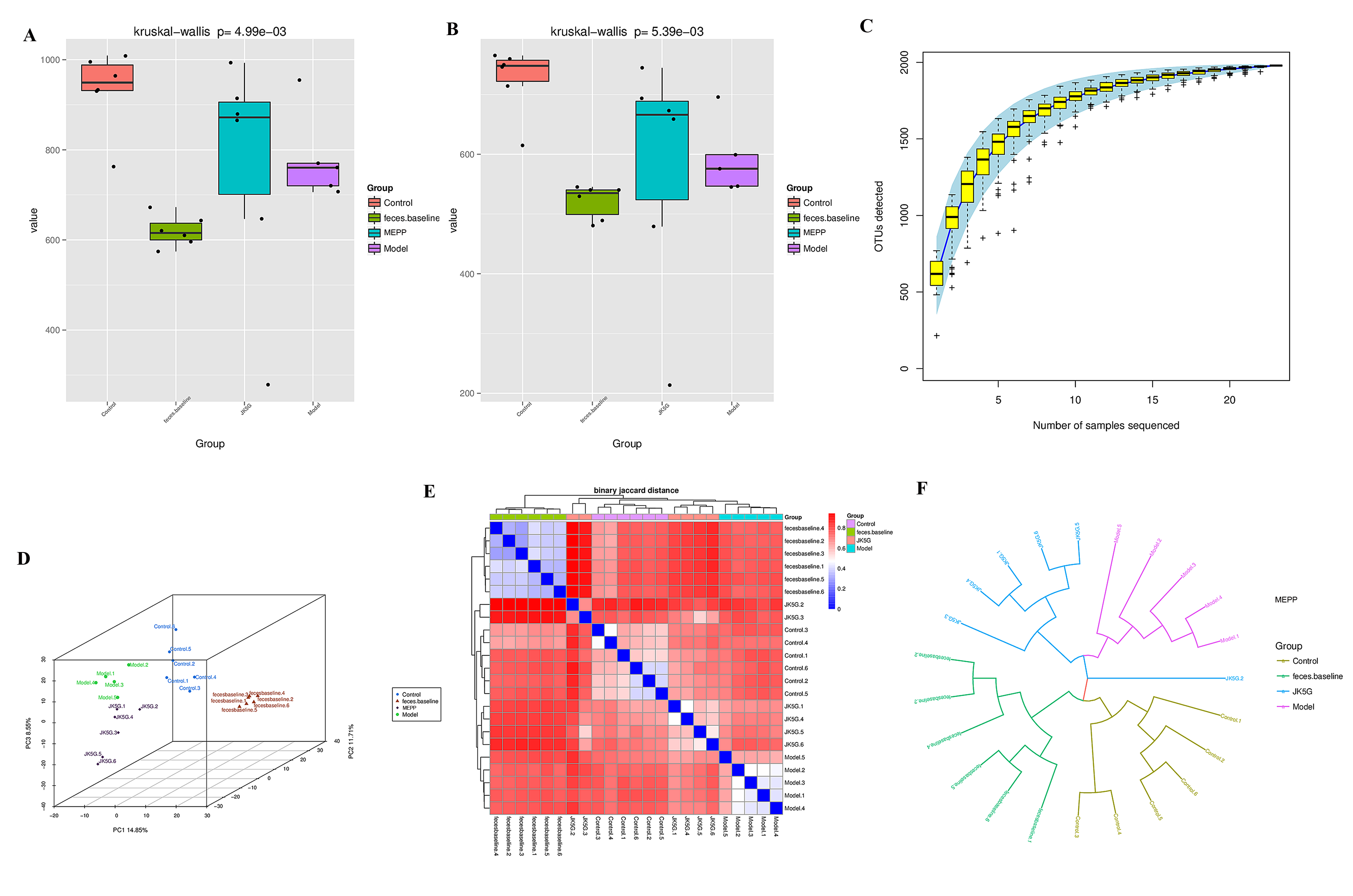

Supplement: Supplementary Figure 1 — Diversity analysis. Alpha diversity analysis (A–C), the horizontal axis is sample size, and the vertical axis is the number of OTUs. If the curve shows a sharp rise, it indicates that the sampling quantity is insufficient as the sampling quantity increases; if the curve tends to flatten, it means that the species in this environment will not increase significantly with the increase of sample size, indicating that the sampling is adequate. Beta-diversity analysis (D–F), the horizontal and vertical axes of the figure represent two eigenvalues that can reflect the variance to the greatest extent. Each point in the figure is a sample, and the same color is the same group. [file Image_1.TIF]

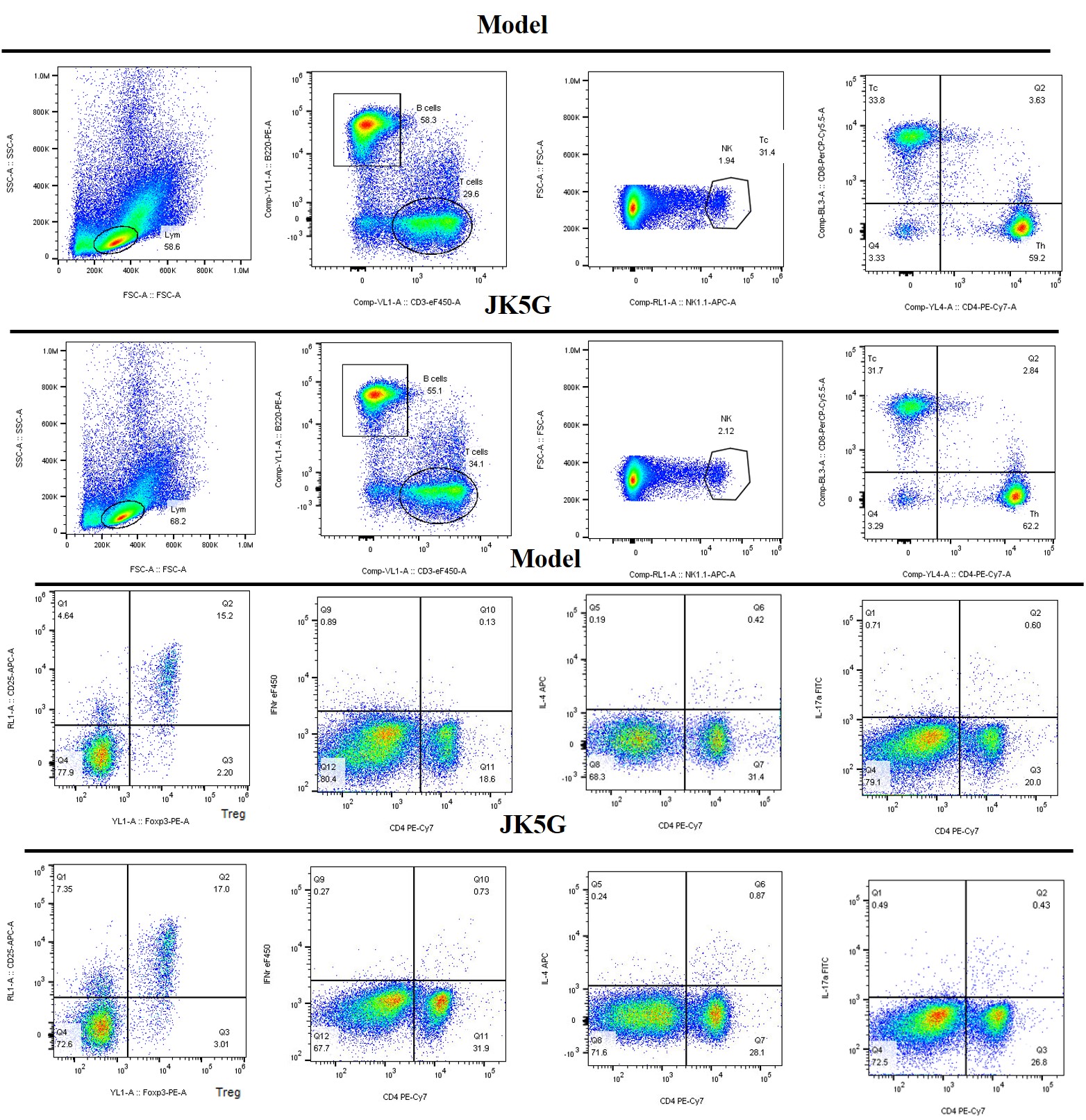

Supplement: Supplementary file 2 [file Image_2.JPEG]

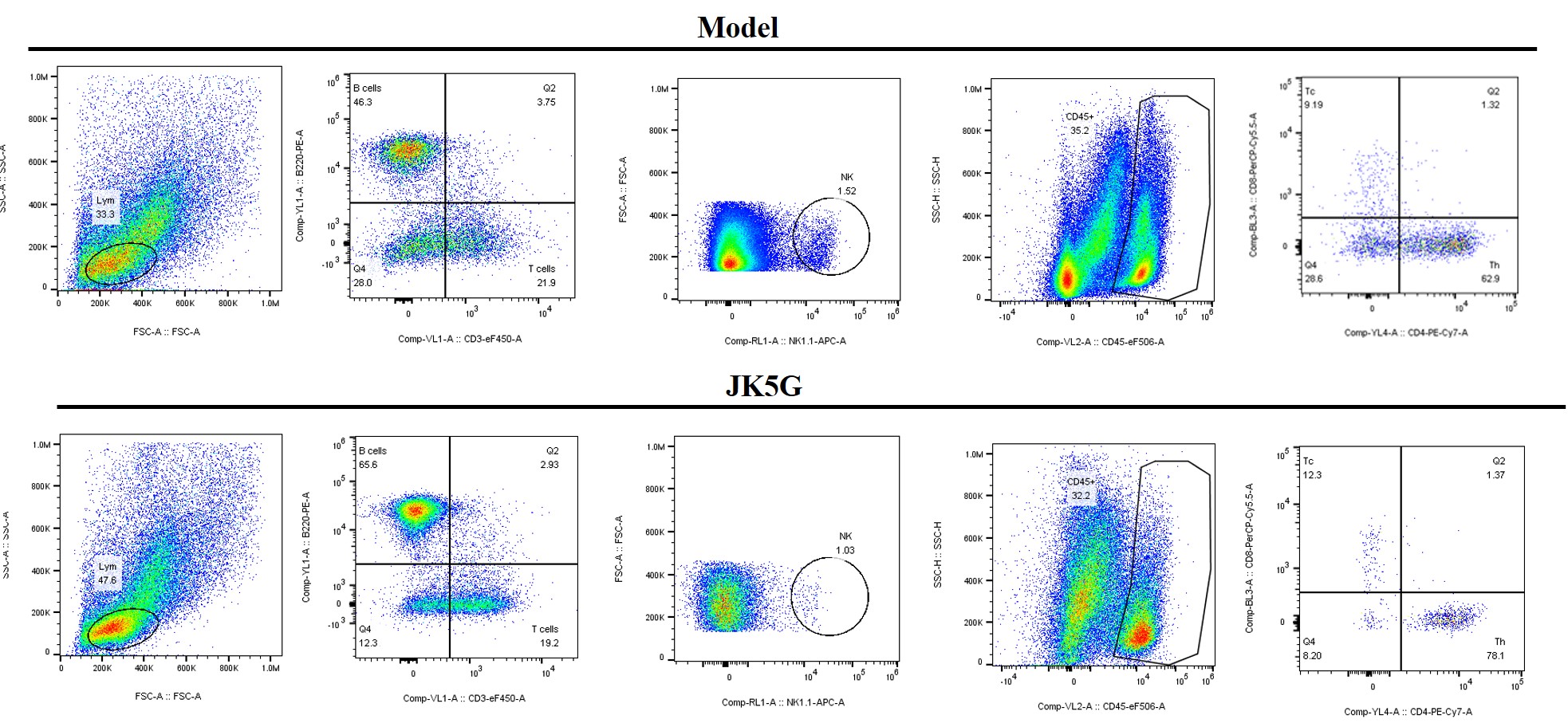

Supplement: Supplementary file 3 [file Image_3.JPEG]
