# Supplementary material for: Inhibitory Effect of a Microecological Preparation on Azoxymethane/Dextran Sodium Sulfate-Induced Inflammatory Colorectal Cancer in Mice
Source: Front Oncol. 2020 Oct 16;10:562189. doi: 10.3389/fonc.2020.562189 (PMC7596756; doi:10.3389/fonc.2020.562189)
Supplement: Supplementary file 4 [file Data_Sheet_1.docx]

**Supplementary Tables**

**Table S1 The antibody information of flow cytometry**

| **Name** | **Company** | **CAS** |
| --- | --- | --- |
| anti-mouse CD4 FITC | Thermo | 11-0042-82 |
| anti-mouse Foxp3 PE | Thermo | 12-5773-82 |
| anti-mouse CD25 APC | Thermo | 17-0251-82 |
| anti-mouse NK1.1 APC | BioLegend | 108710 |
| anti-mouse CD3 eF450 | Thermo | 48-0031-82 |
| anti-human/mouse B220 PE | Thermo | 12-0452-81 |
| anti-mouse CD4 PE-Cy7 | BioLegend | 100528 |
| anti-mouse CD8a PerCP-Cy5.5 | Thermo | 45-0081-82 |
| anti-mouse IL-17A AF488 | BioLegend | 506909 |
| anti-mouse IL-4 APC | Thermo | 17-7041-81 |
| anti-mouse IFNr eF450 | Thermo | 48-7311-82 |

**Table S2A The differential microbiome at phylum and genus levels in Model vs Control**

| Model vs Control | OTU | Test-Statistic | P | TYPE |
| --- | --- | --- | --- | --- |
| phylum | Verrucomicrobia | 8.919 | 2.82E-03 | UP |
|  | Actinobacteria | 4.428 | 3.53E-02 | UP |
| genus | Prevotellaceae_UCG_001 | 8.919 | 2.82E-03 | UP |
|  | Bifidobacterium | 8.919 | 2.82E-03 | UP |
|  | Akkermansia | 8.919 | 2.82E-03 | UP |
|  | Lachnospiraceae_UCG_008 | 8.250 | 4.08E-03 | DOWN |
|  | Ruminococcaceae_UCG_005 | 7.895 | 4.96E-03 | DOWN |
|  | Lachnospiraceae_UCG_001 | 7.857 | 5.06E-03 | DOWN |
|  | Marvinbryantia | 7.639 | 5.71E-03 | UP |
|  | Blautia | 7.569 | 5.94E-03 | UP |
|  | Parasutterella | 7.534 | 6.05E-03 | UP |
|  | Allobaculum | 7.534 | 6.05E-03 | UP |
|  | Erysipelatoclostridium | 7.534 | 6.05E-03 | UP |
|  | Other | 7.500 | 6.17E-03 | DOWN |
|  | Odoribacter | 7.500 | 6.17E-03 | UP |
|  | Parabacteroides | 7.500 | 6.17E-03 | UP |
|  | Candidatus_Arthromitus | 6.227 | 1.26E-02 | DOWN |
|  | Ruminococcaceae_NK4A214_group | 5.711 | 1.69E-02 | DOWN |
|  | [Eubacterium]_coprostanoligenes_group | 5.242 | 2.20E-02 | UP |
|  | Romboutsia | 5.242 | 2.20E-02 | UP |
|  | Escherichia_Shigella | 5.029 | 2.49E-02 | UP |
|  | Bacteroides | 4.800 | 2.85E-02 | UP |
|  | Lachnoclostridium | 4.800 | 2.85E-02 | UP |
|  | Tyzzerella_3 | 4.472 | 3.45E-02 | DOWN |
|  | Prevotellaceae_Ga6A1_group | 4.400 | 3.59E-02 | UP |
|  | Anaeroplasma | 4.370 | 3.66E-02 | UP |
|  | Anaerovorax | 4.225 | 3.98E-02 | DOWN |
|  | Alloprevotella | 4.186 | 4.08E-02 | DOWN |
|  | uncultured | 4.033 | 4.46E-02 | DOWN |
|  | Eisenbergiella | 3.986 | 4.59E-02 | UP |

**Table S2B The differential microbiome at phylum and genus levels in WS vs Model**

| JKG5 vs Model | OUT | Test-Statistic | P | TYPE |
| --- | --- | --- | --- | --- |
| genus | Ruminiclostridium | 7.569 | 5.94E-03 | DOWN |
|  | Prevotellaceae_UCG_001 | 7.534 | 6.05E-03 | DOWN |
|  | Other | 7.500 | 6.17E-03 | UP |
|  | Acetitomaculum | 7.413 | 6.48E-03 | DOWN |
|  | Alloprevotella | 7.171 | 7.41E-03 | UP |
|  | Oscillibacter | 6.717 | 9.55E-03 | DOWN |
|  | Family_XIII_AD3011_group | 6.216 | 1.27E-02 | DOWN |
|  | Lachnospiraceae_FCS020_group | 4.866 | 2.74E-02 | DOWN |
|  | Anaerovorax | 4.368 | 3.66E-02 | DOWN |
|  | Lachnospiraceae_NK4A136_group | 4.033 | 4.46E-02 | DOWN |

**Table S3 The differential pathways in Model vs Control, as well as JKG5 vs Model**

|  | PATHWAY ID | P |
| --- | --- | --- |
| Model vs Control | Caffeine metabolism | 2.82E-03 |
|  | Circadian rhythm - plant | 2.82E-03 |
|  | Fatty acid elongation in mitochondria | 2.82E-03 |
|  | Systemic lupus erythematosus | 2.82E-03 |
|  | Glycosphingolipid biosynthesis - lacto and neolacto series | 5.71E-03 |
|  | Steroid biosynthesis | 5.71E-03 |
|  | Arachidonic acid metabolism | 6.17E-03 |
|  | Carbohydrate digestion and absorption | 6.17E-03 |
|  | Meiosis - yeast | 6.17E-03 |
|  | Steroid hormone biosynthesis | 6.17E-03 |
|  | Ubiquitin system | 6.17E-03 |
|  | African trypanosomiasis | 1.76E-02 |
|  | Amoebiasis | 1.76E-02 |
|  | Biosynthesis of siderophore group nonribosomal peptides | 1.76E-02 |
|  | Pathogenic Escherichia coli infection | 2.12E-02 |
|  | Shigellosis | 2.12E-02 |
|  | Fluorobenzoate degradation | 2.70E-02 |
|  | Bacterial invasion of epithelial cells | 2.77E-02 |
|  | Chagas disease (American trypanosomiasis) | 2.85E-02 |
|  | Pores ion channels | 2.85E-02 |
| JKG5 vs Model | Ether lipid metabolism | 4.41E-02 |
|  | Isoquinoline alkaloid biosynthesis | 4.46E-02 |
|  | Linoleic acid metabolism | 4.46E-02 |
|  | Pathways in cancer | 4.46E-02 |
|  | Photosynthesis | 4.46E-02 |
|  | Photosynthesis proteins | 4.46E-02 |
|  | Polycyclic aromatic hydrocarbon degradation | 4.46E-02 |

**Table S4 The differential metabolites in faeces in Model vs Control, as well as JKG5 vs Model**

|  | **Metabolites** | **Formula** | **VIP** | **P-value** | **log2(FC)** |
| --- | --- | --- | --- | --- | --- |
| Model vs Control | 3,4,5-trihydroxy-6-{[14-methoxy-6-(3-methylbut-2-en-1-yl)-8,17 dioxatetracyclo[8.7.0.0²,⁷.0¹¹,¹⁶]heptadeca-2(7),3,5,11(16),12,14-hexaen-5-yl]oxy}oxane-2-carboxylic acid | C27H30O10 | 2.385 | 1.19E-04 | -2.181 |
|  | 3-Galactosyllactose | C18H32O16 | 2.946 | 1.99E-04 | 1.712 |
|  | Indole | C8H7N | 1.031 | 1.99E-04 | -0.897 |
|  | 2-Dimethylamino-5,6-dimethylpyrimidin-4-ol | C8H13N3O | 2.135 | 9.50E-04 | -3.235 |
|  | PI(14:0/0:0) | C23H45O12P | 1.131 | 1.63E-03 | 1.996 |
|  | 2,5-Dihydro-2,4,5-trimethyloxazole | C6H11NO | 1.014 | 1.72E-03 | -2.035 |
|  | Lenticin | C14H18N2O2 | 3.698 | 1.78E-03 | -1.478 |
|  | 1α,25-dihydroxy-22,23-didehydro-24a,24b,24c-trihomovitamin D3 / 1α,25-dihydroxy-22,23-didehydro-24a,24b,24c-trihomocholecalciferol | C30H48O3 | 4.534 | 2.96E-03 | -2.674 |
|  | 2-(1,2-Diamino-1-propenyl)phenol | C9H12N2O | 2.082 | 3.27E-03 | -3.389 |
|  | Tsugaric acid B | C33H52O5 | 1.072 | 3.31E-03 | -1.091 |
|  | N-Acetyl-7-O-acetylneuraminic acid | C13H21NO10 | 2.831 | 3.34E-03 | 2.557 |
|  | 2,6-Di-tert-butyl-4-ethylphenol | C16H26O | 1.210 | 3.64E-03 | -1.058 |
|  | Mangalkanyl glucoside | C21H38O6 | 1.447 | 3.70E-03 | 1.230 |
|  | 6-deoxyerythronolide B | C21H38O6 | 2.241 | 3.91E-03 | 0.828 |
|  | 1-Stearoylglycerophosphoinositol | C27H53O12P | 4.934 | 3.99E-03 | -0.398 |
|  | TOFA | C19H32O4 | 1.975 | 4.42E-03 | 0.560 |
|  | Fenoldopam | C16H16ClNO3 | 1.270 | 4.48E-03 | 2.296 |
|  | Bassic acid | C30H46O5 | 3.811 | 4.56E-03 | -0.803 |
|  | 2-(acetylamino)-1,5-anhydro-2-deoxy-3-O-b-D-galactopyranosyl-D-arabino-Hex-1-enitol | C14H23NO10 | 1.770 | 4.96E-03 | 1.761 |
|  | (22E,24E)-1alpha,25-dihydroxy-26,27-diethyl-22,23,24,24a-tetradehydro-24a-homovitamin D3 / (22E,24E)-1alpha,25-dihydroxy-26,27-diethyl-22,23,24,24a-tetradehydro-24a-homocholecalciferol | C32H50O3 | 1.656 | 5.46E-03 | -1.459 |
| JKG5 vs Model | Sildenafil | C22H30N6O4S | 1.666 | 1.76E-11 | -5.589 |
|  | Pyridoxamine | C8H12N2O2 | 1.311 | 1.15E-08 | -2.049 |
|  | (9R,13R)-1a,1b-dinor-10,11-dihydro-12-oxo-15-phytoenoic acid | C16H26O3 | 1.060 | 4.27E-08 | -3.605 |
|  | Stevioside | C38H60O18 | 6.213 | 5.41E-08 | -8.688 |
|  | Isoleucyl-Tyrosine | C15H22N2O4 | 2.072 | 2.68E-07 | 2.037 |
|  | Dinorcapsaicin | C16H23NO3 | 1.618 | 2.94E-07 | -2.008 |
|  | 2-Hydroxycinnamic acid | C9H8O3 | 9.478 | 3.28E-07 | 1.667 |
|  | Acevaltrate | C24H32O10 | 1.423 | 3.50E-07 | -3.178 |
|  | (3beta,5alpha,6alpha,7beta,14alpha,22E,24R)-5,6-Epoxyergosta-8,22-diene-3,7,14-triol | C28H44O4 | 2.468 | 7.73E-07 | -2.038 |
|  | Paxilline | C27H33NO4 | 1.120 | 8.97E-07 | -2.830 |
|  | L-Valine | C5H11NO2 | 4.889 | 9.73E-07 | 1.379 |
|  | Hypothiocyanite | CHNOS | 1.945 | 1.19E-06 | 3.466 |
|  | Serotonin | C10H12N2O | 1.783 | 1.20E-06 | 2.445 |
|  | 4-formyl Indole | C9H7NO | 1.885 | 1.22E-06 | 2.301 |
|  | S-(2-Methylbutanoyl)-dihydrolipoamide | C13H25NO2S2 | 1.120 | 1.37E-06 | -1.381 |
|  | Dolichotheline | C10H17N3O | 1.601 | 1.53E-06 | -2.581 |
|  | Tryptophanol | C10H11NO | 1.276 | 1.57E-06 | 2.425 |
|  | Tamoxifen-N-glucuronide | C32H38NO7+ | 1.465 | 1.59E-06 | -3.945 |
|  | 2-Iodophenol methyl ether | C7H7IO | 4.119 | 1.63E-06 | 2.731 |
|  | 5-(sulfooxy)pentanoic acid | C5H10O6S | 1.198 | 1.73E-06 | -1.081 |

**Table S5 Differentially expressed metabolites in serum in Model vs Control, as well as JKG5 vs Model**

|  | **Metabolites** | **Formula** | **VIP** | **P-value** | **log2(FC)** |
| --- | --- | --- | --- | --- | --- |
| Model vs Control | gamma-Eudesmol rhamnoside | C21H36O5 | 1.465 | 3.76E-10 | 2.336 |
|  | 3-hydroxydecanoyl carnitine | C17H33NO5 | 1.520 | 1.21E-09 | 2.149 |
|  | 12-Hydroxydodecanoic acid | C12H24O3 | 1.189 | 4.89E-09 | 2.496 |
|  | 9-Hydroxydecanoic acid | C10H20O3 | 1.490 | 4.96E-09 | 2.129 |
|  | Kynurenine | C10H12N2O3 | 1.326 | 1.43E-08 | -2.212 |
|  | (+/-)-trans- and cis-4,8-Dimethyl-3,7-nonadien-2-ol | C11H20O | 1.728 | 2.15E-08 | 2.146 |
|  | 3-hydroxydodecanoyl carnitine | C19H37NO5 | 1.275 | 3.19E-08 | 2.025 |
|  | O-Ureidohomoserine | C5H11N3O4 | 1.530 | 3.47E-08 | 1.983 |
|  | [3-(4-methoxyphenyl)propoxy]sulfonic acid | C10H14O5S | 1.967 | 6.91E-08 | -1.380 |
|  | 2-(Methylthiomethyl)-3-phenyl-2-propenal | C11H12OS | 2.191 | 7.14E-08 | -2.036 |
|  | 1alpha,25-dihydroxy-19-norvitamin D3 / 1alpha,25-dihydroxy-19-norcholecalciferol | C26H44O3 | 1.229 | 7.29E-08 | -1.822 |
|  | PS(22:2(13Z,16Z)/0:0) | C28H52NO9P | 2.486 | 1.74E-07 | 1.642 |
|  | 11Z-hexadecenoic acid | C16H30O2 | 1.075 | 2.22E-07 | 1.749 |
|  | 24,25,26,27-Tetranor-23-oxo-hydroxyvitamin D3 | C23H34O3 | 1.498 | 2.45E-07 | 1.711 |
|  | Dehydrovomifoliol | C13H18O3 | 1.196 | 3.42E-07 | -1.447 |
|  | Noravicholic acid | C23H38O5 | 1.935 | 4.29E-07 | 1.804 |
|  | Ethyl 3-[(2-furanylmethyl)thio]propanoate | C10H14O3S | 1.055 | 6.45E-07 | -3.470 |
|  | DG(16:0e/18:0/0:0) | C37H74O4 | 1.627 | 8.77E-07 | -0.485 |
|  | Pyranomammea C | C22H28O6 | 1.024 | 1.04E-06 | -0.960 |
|  | 4-(2,6,6-Trimethyl-1-cyclohexenyl)-2-butanol | C13H24O | 1.939 | 1.15E-06 | 1.890 |
| JKG5 vs Model | N-Palmitoyl tyrosine | C25H41NO4 | 1.620 | 6.90E-07 | -1.526 |
|  | Dihydroergotamine | C33H37N5O5 | 3.269 | 1.46E-06 | -1.016 |
|  | Liquoric acid | C30H44O5 | 1.112 | 1.58E-06 | -2.005 |
|  | Sulfacetamide | C8H10N2O3S | 1.102 | 1.60E-06 | 0.480 |
|  | Kolanone | C33H42O4 | 2.017 | 5.02E-06 | -2.084 |
|  | 11-dehydro-TXB2-d4 | C20H28D4O6 | 1.013 | 6.47E-06 | -2.285 |
|  | Methylisopelletierine | C9H17NO | 1.028 | 9.34E-06 | -1.248 |
|  | 3-hydroxyoctanoyl carnitine | C15H29NO5 | 1.147 | 1.08E-05 | -0.784 |
|  | Histidinyl-Histidine | C12H16N6O3 | 1.680 | 1.66E-05 | -0.398 |
|  | Eszopiclone | C17H17ClN6O3 | 6.042 | 2.18E-05 | 0.648 |
|  | L-Urobilin | C33H46N4O6 | 2.285 | 2.50E-05 | -2.053 |
|  | (S)-a-Amino-2,5-dihydro-5-oxo-4-isoxazolepropanoic acid N2-glucoside | C12H18N2O9 | 2.387 | 4.54E-05 | -0.661 |
|  | (9Z)-3-hydroxydodecenoylcarnitine | C19H35NO5 | 1.249 | 4.67E-05 | -0.599 |
|  | Coumestrin | C21H18O10 | 1.513 | 4.85E-05 | 1.422 |
|  | 5,6-Dimethoxysterigmatocystin | C20H16O8 | 1.383 | 6.11E-05 | 1.398 |
|  | 12-Hydroxydodecanoic acid | C12H24O3 | 1.188 | 6.14E-05 | -1.013 |
|  | Secobarbital | C12H18N2O3 | 1.019 | 8.63E-05 | 0.952 |
|  | cis-5-Tetradecenoylcarnitine | C21H39NO4 | 4.381 | 9.37E-05 | -0.832 |
|  | L-Methionine S-oxide | C5H11NO3S | 4.552 | 1.09E-04 | 0.526 |
|  | Flutamide | C11H11F3N2O3 | 1.058 | 1.18E-04 | 0.533 |
